# Antibacterial activities of two potential peptides extracted from *Polistes wattii* Cameron, 1900 (Vespidae: Polistinae) wasp venom collected at Eastern Province, Saudi Arabia

**DOI:** 10.1371/journal.pone.0264035

**Published:** 2022-03-07

**Authors:** Kholoud A. Al-Shammery, Wael N. Hozzein

**Affiliations:** 1 Department of Biology, College of Science, Ha’il University, Ha’il, Saudi Arabia; 2 Botany and Microbiology Department, Faculty of Science Beni-Suef University, Beni-Suef, Egypt; Manonmaniam Sundaranar University, INDIA

## Abstract

Alternatives of conventional antibiotics have become an urgent need to control drug-resistant bacteria. Therefore, search for new antibacterial agents has become a trend in several microbiological and pharmaceutical scientific works. Insects, one of the most successful and evolved species on earth is known to be an effective natural source of several medically useful chemicals including antibacterial agents. There is considerable evidence of using wasp venom against medical ailments in several parts of the world. In this work venom from *Polistes wattii* Cameron, 1900 collected from Eastern Province, Saudi Arabia was evaluated for its antibacterial activities. Such activity was tested against four pathogenic bacteria: two-gram positive *Staphylococcus aureus* (ATCC 25923) and *Streptococcus mutans* (RCMB 017(1) ATCC 25175) and two gram-negative (*Salmonella typhimurium* NCTC 12023 ATCC 14028 and *Enterobacter cloacae* (RCMB 001(1) ATCC 23355). Also, chemical characterization of wasp venom was done using HPLC and two isolated peptides were sequenced. The result indicates the potent anti-microbial effect of the venom against the four tested bacteria. The most sensitive bacteria were *Staphylococcus aureus* (ATCC 25923) and *Streptococcus mutans* (RCMB 017(1) ATCC 25175). The sequence of the two purified peptides indicates that they belong to mastoparan. The study results may pave way to use this wasp venom in future antibiotics especially in controlling skin infection by *Staphylococcus aureus*.

## Introduction

Humans depended mostly on the natural resources for all their needs [1]. Diseases, being the most crucial limiting factor that negated the advancement of human race [2]. Plant derived phytochemicals were the primary resources exploited for human needs [3]. Several plant-derived phytochemicals were investigated for their activity against chronic debilitating diseases and have found to act through multiple pathways including their disease modifying effect and by general mechanisms like antioxidant defense [4, 5]. But, in the present scenario, where the disease-causing agents have attained resistance against the drug, these medicines failed drastically. In such situations, newer alternatives have been searched for in other life forms like animal derived drugs developed from their natural secretions. Animal derived glandular secretions like musk are being traditionally used for medicinal purposes [6]. Salivary secretions, specialized glandular secretions constituting venom, secretions for self-defense in the form of acrid irritant juices; all represents potent sources against human diseases [7]. From an evolutionary point of view-, animal venom forms a very effective group of chemicals that was used to kill and digest prey [8, 9]. Many animals have evolved a wide range of chemical toxins to achieve this purpose [10]. The class Insecta, in particular, utilized a vast array of chemicals in their venom for the purpose of self-defense and predation [11, 12]. Among insects, the order Hymenoptera, which includes ants, bees and wasps are specifically equipped with effective venom and delivery systems which provided them an evolutionary advantage of becoming the most evolved life forms on earth [13, 14]. The chemical analysis of Hymenoptera venom showed the presence of an array of low molecular weight compounds like amino acids, biogenic amines, carbohydrates, small peptides and phospholipids with diverse biological activity [15].

The medicinal use of Hymenoptera venom dates back to ancient Egyptian civilizations, where the use of honeybee venom was common for alleviating arthralgia [16]. For years, immunotherapy was the main objective of medication by insect venom, as the venom enhances the immune defense and increases blood circulation on target sites [17, 18]. Recently, studies have shown that insect venom is active against viruses, fungi, and most importantly drug-resistant bacteria [19].

Drug-resistant bacteria poses a great threat in the form of escalating health expenditure and loss of precious human lives in different parts of the world [20]. Failure of conventional antibiotics against common bacterial infections is the nightmare faced by microbiologists and pharmacologists alike during the current century [21]. Search for newer and potent anti-microbial agents to combat infections is the urgent need of the hour to prevent wide spread infections without specific medications [22]. Very common pathogenic bacteria like *Staphylococcus aureus*, causing bacteremia and infective endocarditis along with soft tissue infections, and *Salmonella typhimurium*, the common cause for food poisoning, are resistant to a wide range of antibiotics available today [23, 24]. The above illustrated facts point to a grave crisis generated by drug-resistant microbes posing life-threatening conditions from common infections and minor injuries [25].

Although, advancements like passive immunization and phage therapy have substituted conventional antimicrobials to a greater extend, medical researchers are still behind exotic sources of novel antibiotics [26, 27]. Several natural sources were screened for antimicrobial activity and among them insects provided promising results in this regard [20, 28]. Recently, several publications highlighted the biological and chemical activities of hymenopteran insects including their antimicrobial activity [29].

Wasps, a hymenopteran insect, produces a venom which is a good source of alternative antibiotic agents [30]. Antimicrobial peptides (AMPs) isolated from wasp venom have shown strong bactericidal activity [31, 32]. Their mode of action depends on eliciting multiple pathways that include destructing the phospholipid bilayer membrane, perturbing cellular metabolism, or by interfering with cytoplasmic signaling. This makes them a safer alternative for human and animal consumption. Moreover, these compounds are highly conserved among the Vespidae family [33].

In Saudi Arabia, several species of Vespidae are reported [34], but their venoms were never characterized and analyzed before for their antimicrobial activity. This work aims at characterizing two peptides isolated from *Polistes wattii* Cameron, 1900 collected from the Eastern Province of Saudi Arabia and to investigate their antimicrobial activity against four multi-drug resistant strains of *Staphylococcus aureus*, *Streptococcus mutans*, *Salmonella typhimurium* and *Enterobacter cloacae* for the development of a probable antimicrobial agents that can overcome drug resistance.

## Material and methods

### Collected materials

**Fig 1:**
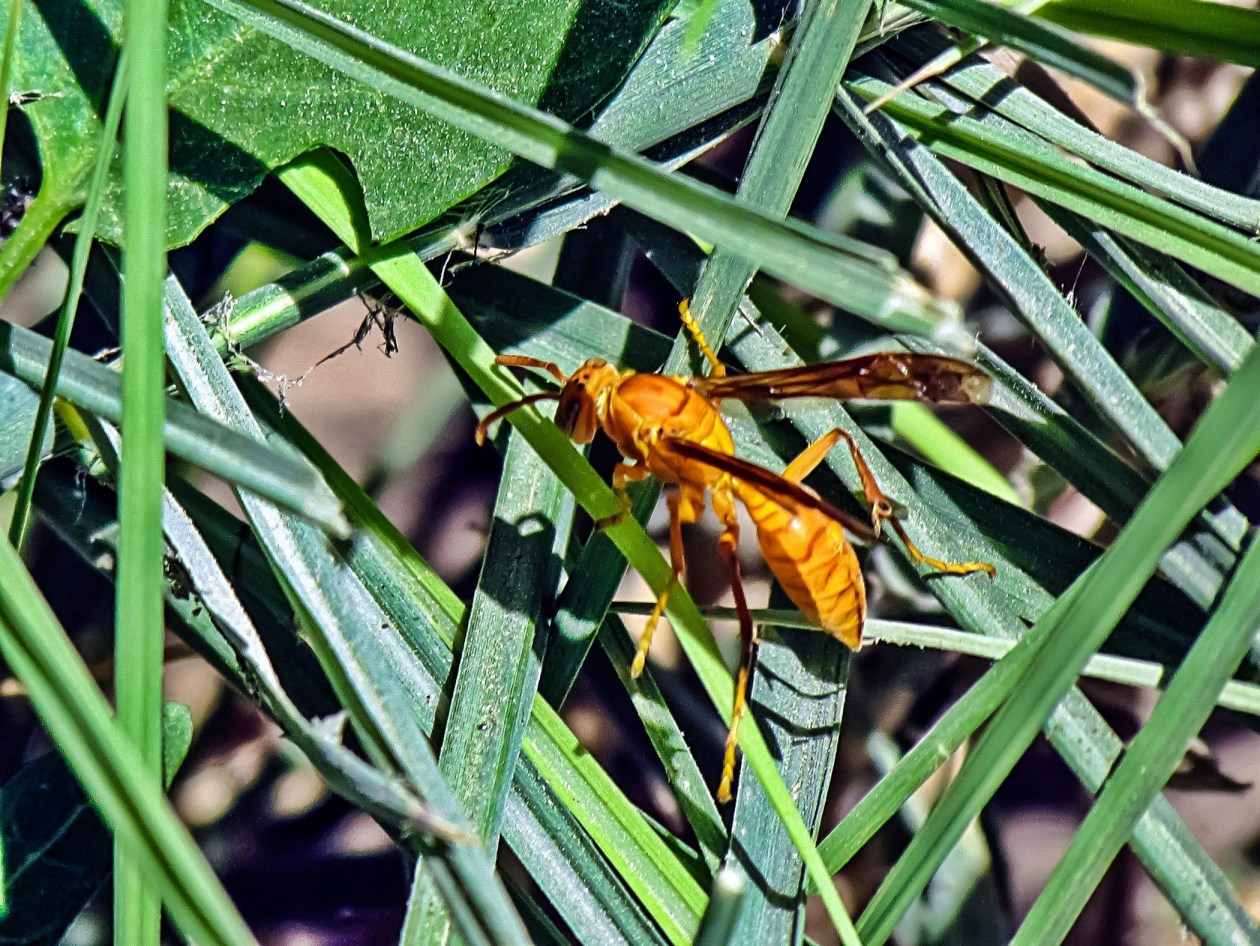
The wasp *Polistes wattii* with its characteristic yellow coloration.

Live specimens of *Polistes wattii* wasps were collected using standard insect swiping net from Al-Ahsa Governorate (25°23′00″N 49°36′00″E) (Fig 1). The specimens were then transferred to the lab and identified according to the keys published by Temreshev, 2018. The venom was collected using an electric screen that was used for collecting honeybee venom with a 6 Volt electric charge [35]. The collected venom was harvested three time a week from electric screen and transferred to Eppendorf tubes containing mixture of 50:50 acetonitrile and water and preserved in refrigerator.

### Antibacterial activity

Four multidrug-resistant bacteria were selected to evaluate the antibacterial activities: two-gram positive bacteria (*Staphylococcus aureus* (ATCC 25923) and *Streptococcus mutans* (RCMB 017(1) ATCC 25175) and two gram-negative (*Salmonella typhimurium* NCTC 12023 ATCC 14028 and *Enterobacter cloacae* (RCMB 001(1) ATCC 23355). Agar well diffusion method was used to demonstrate the antibacterial effect of the wasp venom against the selected microbes [36]. The selected strains of the bacteria were uniformly inoculated on to petri dishes containing nutrient agar media. Wells were made on the plates using a sterile 7 mm cork-borer and 100 ul of diluted wasp venom were poured into each well. The dilutions of 5, 2.5, 1.25, 0.75 mg/ml of wasp venom were selected for the present study and the plates were incubated for 24 h at 37°C [37]. The zone of inhibition of bacterial growth was measured using calipers at the end of the incubation [20]. Each experiment was carried out in triplicates for each concentration and organism.

### Chemical analysis

Wasp venom compounds were isolated using HPLC under a specific column (Vydac® 218TP C18 HPLC Columns, Avantor) with unique selectivity for small peptides. The peptides were collected after a period of 30 min runtime at every minute [38]. Certain pure peptide fractions were then transferred to Porton LF3000G protein sequencing machine to get the amino acid sequence [39]. Chemoffice (chem draw) and Discovery Studio software were used to visualize the selected peptide chemical orientation and 3D shape.

### Statistical analysis

One way ANOVA was done between the different venom concentrations for each bacterial pathogen and the mean zone of inhibition was done using IBM SPSS ver.22 followed by post Hoc Tukey’s test were done to evaluate the differences between the different concentrations. Matrix cluster analyses using two-way single linkage Euclidian distance was made using SYSTAT version 13, from Systat Software, Inc., San Jose, CA, USA, www.sigmaplot.com to show the degree of antimicrobial activity of wasp venom for each pathogenic species [20].

## Results

**Fig 2:**
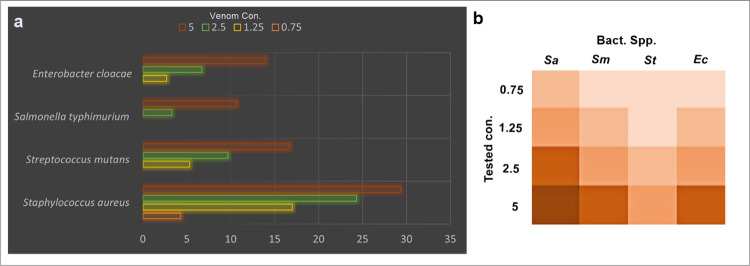
a. Minimum inhibitory concentrations of wasp venom towards certain strains of gram-positive and gram-negative bacteria; b. the heat map that represents the effect of venom on each bacterial species where the darker color indicates the highest effect.

The present work illustrates the antagonistic activity of different *Polistes wattii* wasp venom (PWWV) concentrations to the spectrum of gram-positive and gram-negative human pathogenic bacteria. The result of the agar well diffusion method showed a concentration dependent inhibition of the pathogenic agents (Table 1; Fig 2). All tested bacteria were inhibited by wasp venom with different degrees: the highest inhiation is shown by *Staphylococcus aureus* under the highest concentration 29.3±1.5 while the lowest concentration shows no effect to *Streptococcus mutans*, *Salmonella typhimurium*, and *Enterobacter cloacae* (Fig 2). Matrix cluster analyses produce a heat map that represents each concentration effect on the target bacterial species (Fig 2).

**Table 1:** Antimicrobial activity indicated as inhibition zone in (mm) of different wasp venom concentrations against selected pathogens.

Venom concentration (mg/ml)	Zone of inhibition (mm)			
	Gram-positive bacteria		Gram-negative bacteria	
	*Staphylococcus aureus*	*Streptococcus mutans*	*Salmonella typhimurium*	*Enterobacter cloacae*
**0.75**	4.3±0.9	Na	Na	Na
**1.25**	17.0±1.4	5.3±1.3	Na	2.7±0.7
**2.5**	24.3±1.3	9.7±1.9	3.3±0.7	6.7±1.2
**5**	29.3±1.5	16.7±1.8	10.7±1.9	14.0±1.7

The statistical analysis indicates that there is a significant difference between mean inhibition zone of the different venom concentrations with a P = 0.03. the post hoc Tukey’s test showed a significant difference between each venom concentration (Table 2).

**Table 2:** Overall results of ANOVA test, including non-significant ranges.

Venom concentration (mg/ml)	Mean	Standard error	Non-significant ranges
0.75	1.07	0.3	a
1.25	6.25	0.27	b
2.5	11	0.8	c
5	17.6	1.1	d

**Fig 3:**
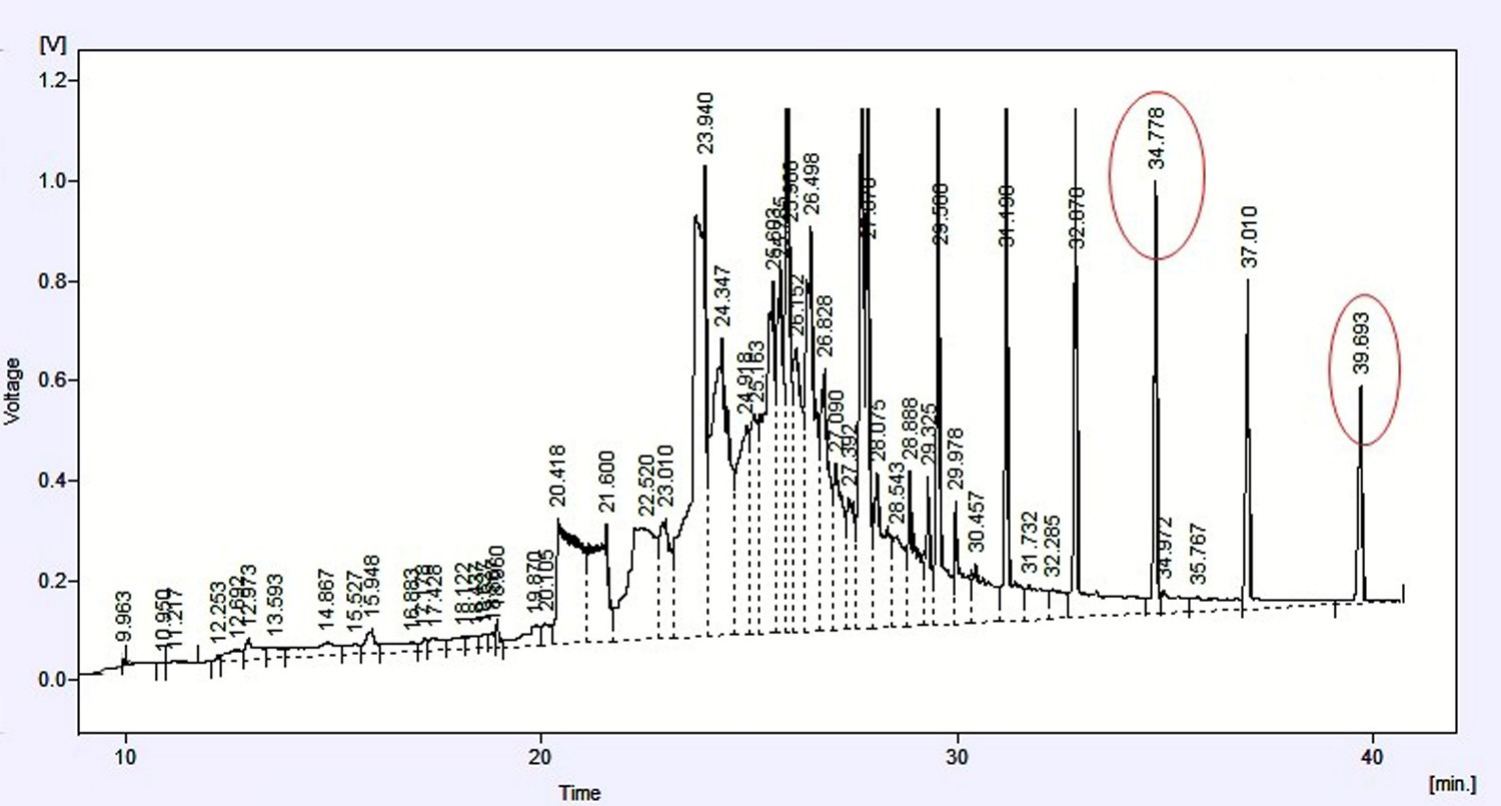
HPLC profile of *Polistes wattii* venom the two red circles indicated the two targeted peptides the chose for sequences.

**Fig 4:**
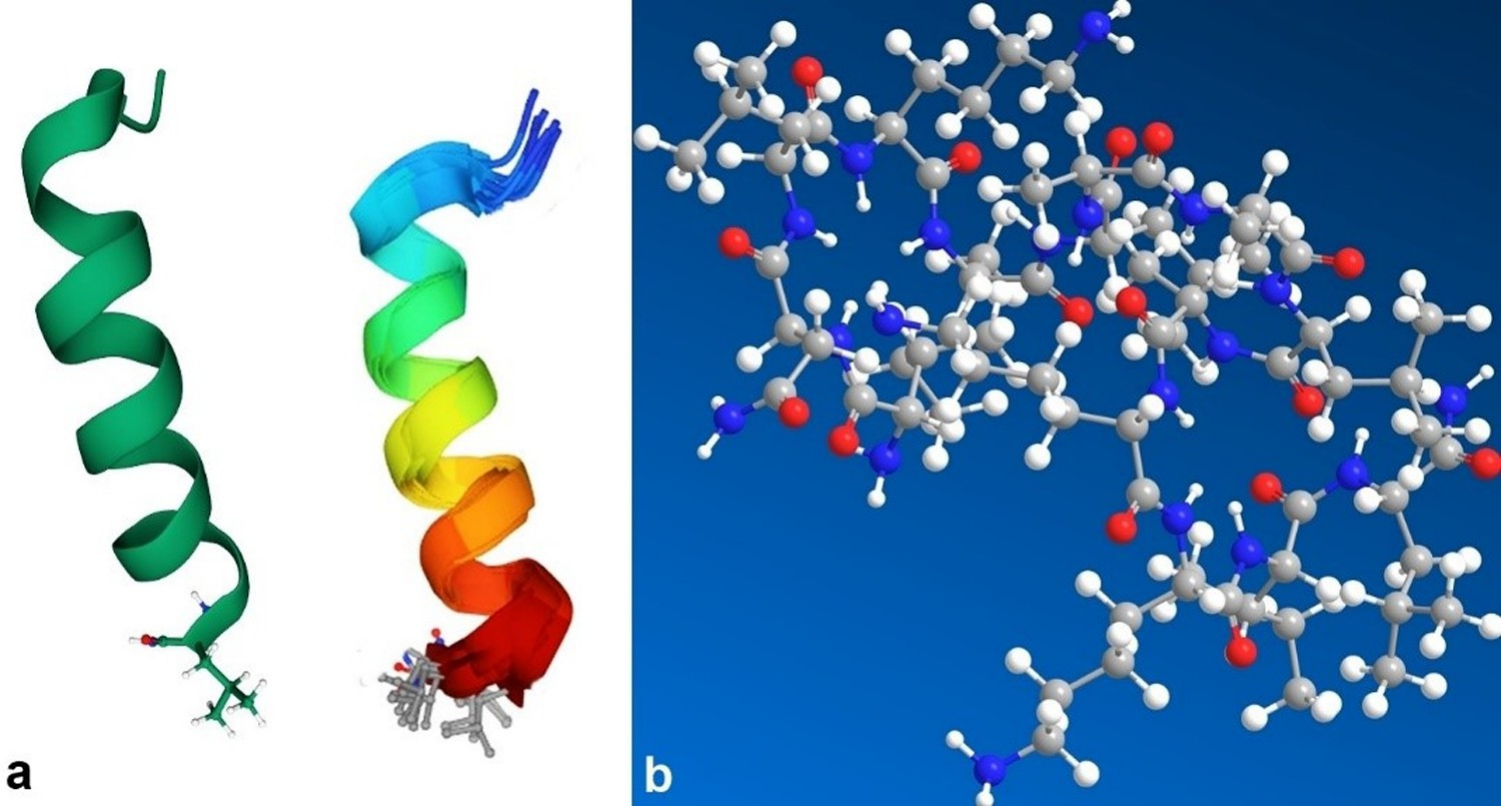
a. Discovery Studio software diagram represents the 3D orientation of mastoparan showing the spiral shape; b. Chem draw diagram which represents the molecular configuration of mastoparan: gray atoms represent Carbon, blue atoms represent Nitrogen, red atoms represent Oxygen and white atoms represent Hydrogen.

The results of HPLC analysis of the venom with the separated venom components are shown in Fig 3. From the literature, the effective small peptides were targeted as the main bioactive compounds that can produce antimicrobial activity so two pure peptides that isolated at retention time 34.778 and 39.693 were sequenced to identify their identity. The sequence result shows that the two peptides have belonged to Mastoparan (a group of toxic peptides that are common in wasp venoms) (Table 3). The two new peptides were given the Acronym of MP-PW1 and MP-PW2 where the MP represents the peptide group mastoparan and the PW represents the wasp species *Polistes wattii*. The chemical drawing software indicated the spiral shape 3D dimension of the isolated peptide that has great ability to disintegrate the phospholipid bilayer of bacterial cells (Fig 4).

**Table 3:** Amino acid sequences of tested mastoparan family of peptides.

Acronym	Retention time (min.)	Sequence
**MP-PW1**	34.778	Ile-Asn-Leu-Lys-Ala-Leu-Ala-Ala-Leu-Ala-Met-Lys-Ile-Leu-NH2
**MP-PW2**	39.693	Ile-Asn-Arg-Lys-Ala-Leu-Ala-Ala-Leu- Met-Met -Lys-Leu-Leu-NH2

## Discussion

The drug-resistant microbial infection poses the most perilous issue in health sector due to the escalating health costs and loss of human resources [40]. World is now facing pandemic attacks from ‘superbugs’ that are resistant to almost all the known antibiotics in use today [41]. Researches ramified in varied related streams like phytomedicine, ethnomedicine and nanomedicine, which searched for effective antimicrobials that could replace the existing antibiotics or could potentiate their action so as to curb the menace created by multidrug resistant varieties of microbes [42–45]. Though succeeded to a certain extent, these remedies largely failed to provide a leap in the medical armamentarium of antibiotic agents [46]. The search has now extended to other natural sources like the venom derived from reptiles and insects. Among these the members of the class insecta evolved with much unique venom profile and diverse biogenic activities. In insects, wasps, belonging to the family of Vespidae distributed all around the world and with more than 5000 species has been the center of attraction owing to its diverse pharmacological activities. Wasp venom is a complex mixture of chemicals containing proteins, peptides, enzymes and small molecules. The common peptides isolated from the wasp venom are mastoparan, eumenitin, eumenitin-R, rumenitin-F, EpVP, decoralin and anoplin [47]. The enzymes, on the other hand included hyaluronidase, α-glucosidase, phosphatase, phospholipase A2 and phospholipase B [48]. In this regard, the variety of antimicrobial, anticancer, neuroprotective anti-oxidant and anti-inflammatory activities exhibited by wasp-derived- peptides are well established. The bioactive peptides derived from PWWV was investigated for its potential to target drug-resistant microbes in the present study. Present study investigated the venom at doses of 5, 2.5, 1.25 and 0.75 mg/ml against the multidrug-resistant bacterial species of *Staphylococcus aureus*, *Streptococcus mutans*, *Salmonella typhimurium* and *Enterobacter cloacae*. The results showed a statistically significant growth inhibition of Wasp venom on the selected microorganisms. Thus, the four selected organisms were inhibited by PWWV with a very predominant and dose dependent activity against Staphylococcus aureus. In earlier studies, the mastoparan-c peptide isolated from *Vespa cabro* venom also showed activity against drug resistant gram-positive and gram-negative microorganisms [49]. PWWV also showed the presence of two mastoparan peptides in HPLC analysis which were sequenced in the present study. Thus, PWWV forms a promising substitute for old and conventional sources of antimicrobial drugs [50]. The presence of wide ranges of antimicrobial peptides in wasp venom encourages the study of more wasp species from around the world to isolate these chemicals and evaluate their antagonistic activity against common multi-drug-resistant bacterial species [51].

Present work showed highest activity against *Staphylococcus aureus*, which is a known notorious pathogen causing skin and respiratory tract infections and other life-threatening conditions like infective endocarditis, toxic shock syndrome, scalded skin syndrome, osteomyelitis, necrotizing fasciitis and necrotizing pneumonia [52, 53]. The versatility and virulence of Staphylococcal infections are attributed to a variety of virulence factors encoded in its genes. for its pathogenicity for human skin [54], *Streptococcus mutans* is a member of the natural flora of human oral cavity mostly dwelling on dental plaques and on biofilms over dental surfaces and is considered one of the common etiological agents for dental caries [55]. *Salmonella typhimurium*, is considered the principal cause for food poisoning and accounts for 3 million deaths in endemic zones annually. It poses a huge impact on the health expenditure of several nations owing to its endemicity and its capability to cause gastroenteritis which is considered a major factor responsible for under 5 years’ mortality among children [56, 57]. *Enterobacter cloacae* is a common Gram-negative facultative, anaerobic, non-sporing bacterium of human gut which gained clinical significance recently owing to its capability to cause opportunistic and nosocomial infections in patients under mechanical ventilation [58, 59]. The four species show quite different responses to the range of diluted concentrations of PWWV. Such outcomes came compatible with other works concerning antipathogenic activity of different wasp venoms throughout the world. Various pathogens show great variation in their response to the venoms [33, 37, 57, 60, 61]. *Staphylococcus aureus* shows highest sensitivity to PWWV even at a very low concentration. This result makes this a potent candidate to be developed as a future drug resistant anti-streptococcal agent. As a common skin pathogen and a predominant species causing soft tissue infections, use of PWWV as a topical agent also could be considered. The incorporation of PWWV into oral toiletries may also be beneficial to contain *Streptococcus mutans* and its propensity to cause oral infections. In contrast, the *Enterobacter cloacae* show less sensitivity to PWWV; this could be due to the exposure of these agents to “antibiotic-pollution” leading to attainment of resistance to multiple drugs [62]. The venom of the very common wasp species *Vespa orientalis* also shows very few effects on *Enterobacter cloacae* [37].

The chemical analysis of PWWV showed similarity to other wasp species of the family Vespidae [63]. The sequenced peptides through this study are very similar to those identified from family Vespidae with an aspartate residue in the second position and very few amino acids substitutions [64]. As all identified mastoparan the molecular orientation of the two identified antimicrobial peptides MP-PW1 and MP-PW2 have a α-helical shape with 14 amino acid residues and an amide group at the C-terminus. Being a small molecule, it has an ability to penetrate the bacterial cell wall easily. AMPs is a versatile molecule that typically acts through a variety of mechanisms of action, which can range from direct interactions and membrane destabilization to intracellular targets [65, 66]. The chemical mode of action of antagonistic activity of these peptides needs to be reviewed and studied for help in developing very effective pharmaceutical final products that will be the antibiotics of the future.

Further works are needed to compare the sequences of different AMPs collected from other wasp species around the world. This will help in synthesizing very effective artificial peptides that could be more effective as antimicrobial agents. The way is still far from getting a complete understanding of such group of new antibiotics till found them on the market, but no doubt they will form a part of our future medicines.

## Supporting information

S1 File Inhibition zone induced by wasp venom using well diffusion method on the left side the control using solvent only and on the right side the venom application (all photos represent the high concentration of the venom): a. *Staphylococcus aureus*; b. *Streptococcus mutans*; c. *Salmonella typhimurium*; d. *Enterobacter cloacae*.
(DOCX)
