# Supplementary material for: Antibacterial activities of two potential peptides extracted from Polistes wattii Cameron, 1900 (Vespidae: Polistinae) wasp venom collected at Eastern Province, Saudi Arabia
Source: PLoS One. 2022 Mar 7;17(3):e0264035. doi: 10.1371/journal.pone.0264035 (PMC8901064; doi:10.1371/journal.pone.0264035)
Supplement: S1 File — Inhibition zone induced by wasp venom using well diffusion method on the left side the control using solvent only and on the right side the venom application (all photos represent the high concentration of the venom): a. Staphylococcus aureus; b. Streptococcus mutans; c. Salmonella typhimurium; d. Enterobacter cloacae. (DOCX) [file pone.0264035.s001.docx]

**Supplementary material 1**: Inhibition zone induced by wasp venom using well diffusion method on the left side the control using solvent only and on the right side the venom application (all photos represent the high concentration of the venom): a. *Staphylococcus aureus*; b. *Streptococcus mutans*; c. *Salmonella typhimurium*; d. *Enterobacter cloacae*

**
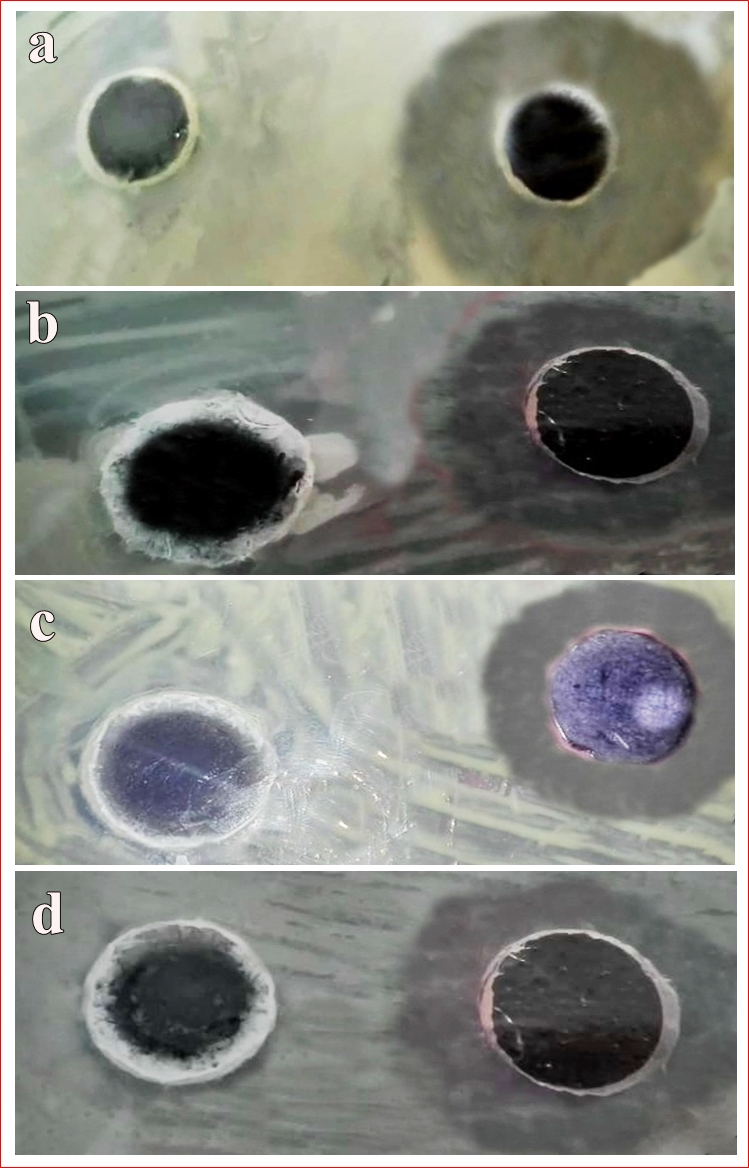
**
